# Breast self-examination practice among women in Africa: a systematic review and Meta-analysis

**DOI:** 10.1186/s13690-021-00671-8

**Published:** 2021-08-21

**Authors:** Wubareg Seifu, Liyew Mekonen

**Affiliations:** 1 Department of Epidemiology, College of Medicine and Health Science, School of Public Health, Jigjiga University, Jigjiga, Ethiopia; 2 Department of Reproductive Health, College of Medicine and Health Science, School of Public Health, Jigjiga University, Jigjiga, Ethiopia

**Keywords:** Breast self-examination, Prevalence, Women, Africa, Systematic review, Meta-analysis

## Abstract

**Background:**

In resource limited countries breast self-examination has been recommended as the most appropriate method for early detection of breast cancer. Available studies conducted on breast self-examination practice in Africa currently are inconsistent and inclusive evidences. On top of that the available studies are unrepresentative by regions with small sample size. Therefore, this systematic review and meta-analysis were conducted to summarize and pool the results of individual studies to produce content level estimates of breast self-examination practice in Africa.

**Methods:**

A systematic review and meta-analysis were done among studies conducted in Africa using Preferred Item for Systematic Review and Meta-analysis (PRISRMA) guideline. Studies were identified from PubMed, Google Scholar, HINARI, EMBASE, CINAHL, Cochrane, African Journals Online and reference lists of identified prevalence studies. Unpublished sources were also searched to retrieve relevant articles. Critical appraisal of studies was done through Joanna Briggs Institute Meta-Analysis of Statistics Assessment and Review Instrument (JBI-MAStARI). The meta-analysis was conducted using STATA 13 software. Heterogeneity was assessed using I^2^ statistics while publication was assessed through funnel plot. Forest plot were used to present the pooled prevalence with a 95% confidence interval (CI) using the random effect model.

**Results:**

In this meta-analysis 56 studies were included with a total of 19, 228 study participants. From the included studies 25(44.64%) were from West Africa, 22(39.29%) East Africa, 5(8.93%) North Africa, 3(5.36%) Central Africa and 1(1.79%) South Africa. The overall pooled prevalence of ever and regular breast self-examination practice in Africa was found to be 44.0% (95% CI: 36.63, 51.50) and 17.9% (95% CI: 13.36, 22.94) respectively. In the subgroup analysis there was significant variations between sub regions with the highest practice in West Africa, 58.87% (95 CI%: 48.06, 69.27) and the lowest in South Africa, 5.33% (95 CI%: 2.73, 10.17).

**Conclusion:**

This systematic review and meta-analysis revealed that breast self-examination practice among women in Africa was low. Therefore, intensive behavioral change communication and interventions that emphasize different domains should be given by stakeholders.

**PROSPERO registration number:**

CRD42020119373.

**Supplementary Information:**

The online version contains supplementary material available at 10.1186/s13690-021-00671-8.

## Background

Breast cancer is the most commonly diagnosed cancer in women and the leading cause of cancer death worldwide, with an estimated 1.7 million new cases and 521,900 deaths in 2012 compared to 1.38 million new cases and 458,000 deaths in 2008 [1–3]. Based on Global Cancer Observatory (GLOBOCAN) estimates, about 14.1 million new cancer cases and 8.2 million deaths occurred in 2012 worldwide [3].

The burden of cancer has shifted to low and middle income countries (LMIC), which currently account for about 57% of cases and 65% of cancer deaths worldwide [3]. Nearly 60% of deaths due to breast cancer occur in LMIC [4]. Recent global cancer statistics indicated that breast cancer incidence is rising at a faster rate in populations of LMIC [5, 6]. The age-standardized incidence rates of breast cancer incidence for the year 2012 in Africa regions were estimated as; 30.4 in eastern Africa (per 100,000 women per year), 26.8 in middle Africa, 38.6 in western Africa, 38.9 in southern Africa and, 33.8 in sub-Saharan Africa [1, 7, 8]. Morbidity and mortality of breast cancer is emerging as a major public health concerns in many LMICs [9]. The lifetime risk of a woman getting breast cancer is 1 in 10 [10]. The main reason for increasing mortality is mainly due to late diagnosis of the disease and lack of feasible early screening programs [11, 12].

Early diagnosis and survival improvement of breast cancer is a top priority to reduce the increasing mortality rate, projected to reach 112, 000 deaths in 2040 [13]. Detecting and preventing breast cancer at an early stage through feasible screening approaches is a very essential recommendation to meet sustainable development goal (SDG) 3.4 by 2030 [14]. Breast cancer is curable if detected early through screening and early diagnosis by breast self-examination (BSE), clinical breast examination (CBE), and mammography [15]. Despite the existence of controversies about the effectiveness breast self-examination in reducing mortality and morbidity [16–18], the technique remains an important approach for early detection mainly in low and middle-income countries where access to diagnostic and curative facilities may be problematic [19, 20].

Breast self-examination practice is the recommended approach in developing countries because it is easy to perform, feasible, convenient, safe and requires no specific equipment and set up [21–23]. Despite this recommendation, available studies conducted on breast self-examination practice in Africa currently are inconsistent and inclusive to inform and direct stakeholders. On top of that the available reviews lacks comprehensives since they were limited to country level with small sample size and high heterogeneity in their results. Therefore, this systematic review and meta-analysis were conducted to summarize and pool the results of individual studies to produce continent level estimates of breast self-examination practice in Africa. The finding of the study will be contributing for designing feasible strategies, polices and guidelines to improve breast self-examination practice and also to fight against breast cancer among women in Africa.

## Methods

### Search strategy

This systematic review and meta-analysis was reported according to the Preferred Reporting Items for Systematic Reviews and Meta-Analysis (PRISMA) statement guideline. Pertinent published articles were searched in the following electronic bibliographic databases: PubMed, EMBASE, Science Direct, HINARI, Google scholar, WHO Global Index Medicus and African Journals Online (AJOL) were searched to retrieve all available studies. In addition, cross-references of included studies were hand-searched as well to access additional relevant articles that may have been missed in the search. We used Medical Subject Heading (MeSH) and keywords to identify relevant studies from the respective database. The search terms were used separately and together using Boolean operators “OR” or “AND”. The key word of search strategy used to retrieve relevant articles was as follows: (((“Breast Self Examination”[MeSH Terms] OR “self examination breast” OR “early detection of breast cancer” OR “breast cancer screening”])) AND (“health knowledge, attitudes, practice”[MeSH Terms]])) AND (“women”[MeSH Terms] OR “Girls” OR “Woman” OR “female” OR “females” OR “Reproductive age women” OR “reproductive aged women”])) AND (“Africa”[MeSH Terms] OR (((“Africa central”] OR “Africa eastern” OR “Africa southern” OR “Africa western” OR “Africa northern”))). The software EndNote version X8 (Tomson Reuters, New York, NY) was used to manage references and remove duplicated references. All articles published up to June 30, 2020 in English language were included in the review if fulfilled the eligibility criteria. This systematic review and meta-analysis was registered in PROSPERO with a registration number; http://www.crd.york.ac.uk/PROSPERO/display_record.asp?ID=CRD42020119373

### Eligibility criteria

#### Inclusion criteria

##### Study design

Observational (case-control, cohort, cross-sectional) studies reporting breast self-examination practice among women in Africa were included.

##### Study area

Only studies conducted in Africa continent were included.

##### Language

Studies that were conducted only in English language were included.

##### Publication status

Both published and unpublished articles were included.

##### Publication period

All publication reported up to June 30, 2020 were included.

##### Population

Studies which were conducted among women in Africa.

##### Outcome

Women who have ever/regularly performed breast self-examination for detection of breast abnormalities and lumps.

### Exclusion criteria

Studies were excluded if they were not primary studies (such as review articles, conference abstract, editorials, case reports am expert opinion). Moreover, studies not reporting the outcome variable, published in any language other than English, author contact not replied within 3 weeks, and qualitative studies were excluded.

### Study selection

First, articles were assessed for inclusion through a title and abstract review by two independent reviewers. Second, potentially-eligible studies were undergoing full-text review to determine if they satisfy the criteria set for inclusion. We did a full-text review in duplicate and clearly document reasons for inclusion and exclusion. Finally, data were extracted from all articles that meet the inclusion criteria. The data extraction form was pre-tested with 3–5 eligible studies. The practice of breast self-examination was extracted if only reported and/or estimated based on experts’ opinion or previously published studies or guidelines. In case of incomplete data, the corresponding author(s) were contacted to find full information. Disagreement and unclear information in the selection of articles being included in the review were resolved through discussion and consensus.

**Fig. 1:**
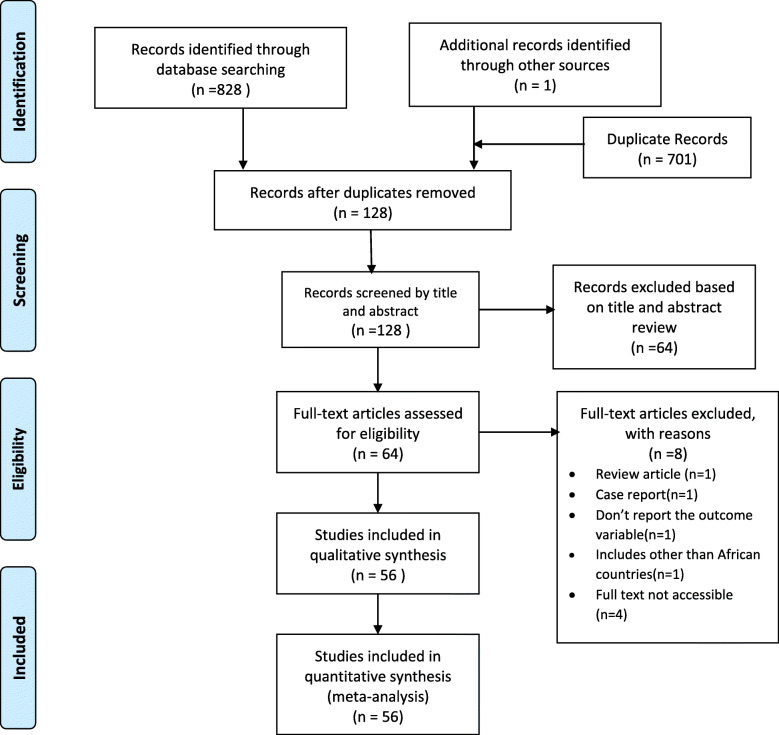
Flow chart diagram describing selection of studies for a systematic review and meta-analysis of prevalence of breast self-examination in Africa, 2020 (identification, screening, eligible and included studies)

In our search we identified 829 articles from different electronic databases. From these, 701 were found duplicate records and removed from the review. Fifty-one and thirteen articles were excluded by reviewing the title and abstract respectively. After a full review of articles, eight were excluded. Three studies didn’t fulfill the inclusion criteria, one articles fail to report the outcome variables and four articles unable to get access to the full articles. Finally, 56 were found to be eligible and included in this meta-analysis (Fig. 1).

### Outcome measures

The primary outcome variable of this study is breast self-examination practice (ever/regular) among women in Africa. Ever breast self-examination practice is defined as a woman who performed breast self-examination irregularly for the purpose of detecting and feeling any abnormal swelling or lumps in their breast tissue which was assessed through interview administered questionnaires. Regular breast self-examination practice when a woman performed breast self-examination during menses once per month which was assessed through interview administered questionnaires.

### Quality assessment

Quality assessment was conducted based on Hoy 2012 tool by two reviewers using 10 criteria addressing internal and external validity [24]. The items included the following ten parameters: (1) representation of the population, (2) sampling frame, (3) methods of participants’ selection, (4) non-response bias, (5) data collection directly from subjects, (6) was an acceptable case definition used, (7) was tool shown reliability and validity, (8) was the same mode of data collection used, (9) was the length of prevalence period appropriate, and (10) were the numerator and denominator appropriate. Each item was assessed as either low or high risk of bias. Unclear was regarded as high risk of bias. In this study, each of the ten parameters in the risk of bias tool was allocated an equal weight. Therefore, the overall assessment of bias was ultimately dependent on the number of high risk parameters out of the ten parameters in the included studies. Finally, the overall risk of bias was graded as high quality (≤ 2), medium quality [3, 4], and low quality (≥ 5) based on the number of high risk parameters per individual studies (Table 1).

**Table 1:** Risk of bias/quality assessment of included studies using the Hoy 2012 tool

Study	Representation	Sampling	Random selection	Non response bias	Data collection	Case Definition	Reliability and validity of study tool	Method of data collection	Prevalence period	Numerator and denominator	Risk of Bias
Birhane et al.	Low risk	Low risk	Low risk	Low risk	Low risk	Low risk	Low risk	Low risk	Low risk	Low risk	Low risk
Obaji et al.	Low risk	High risk	High risk	Low risk	Low risk	High risk	Low risk	Low risk	Low risk	Low risk	Moderate risk
Onwere et al.	High risk	High risk	High risk	Low risk	Low risk	Low risk	Low risk	Low risk	Low risk	Low risk	Moderate risk
Abay et al.	Low risk	Low risk	Low risk	Low risk	Low risk	Low risk	Low risk	Low risk	Low risk	Low risk	Low risk
Minasie A et al.	Low risk	Low risk	Low risk	Low risk	Low risk	Low risk	Low risk	Low risk	Low risk	Low risk	Low risk
Abdel Fattah, M et al.	High risk	High risk	High risk	Low risk	Low risk	Low risk	Low risk	Low risk	Low risk	Low risk	Moderate risk
Abeje et al.	High risk	Low risk	High risk	Low risk	Low risk	Low risk	Low risk	Low risk	Low risk	Low risk	Low risk
Birhane K et al.	Low risk	Low risk	Low risk	Low risk	Low risk	Low risk	Low risk	Low risk	Low risk	Low risk	Low risk
Carlson-Babila Sama et al.	High risk	Low risk	Low risk	Low risk	Low risk	Unclear	Low risk	Low risk	Low risk	Low risk	Low risk
Kasahun AF	Low risk	Low risk	Low risk	Low risk	Low risk	Low risk	High risk	Low risk	Low risk	Low risk	Low risk
Dagne AH et al.	Low risk	Low risk	Low risk	Low risk	Low risk	Low risk	Low risk	Low risk	Low risk	Low risk	Low risk
Dadzi R, Adam A	Low risk	Low risk	High risk	Low risk	Low risk	Unclear	Low risk	Low risk	Low risk	Low risk	Low risk
Gwarzo, UMD et al	Low risk	Low risk	Low risk	Low risk	Low risk	Low risk	Low risk	Low risk	Low risk	Low risk	Low risk
Isara, A. R. and Ojedokun, C. I	Low risk	Low risk	Low risk	Low risk	Low risk	Unclear	Low risk	Low risk	Low risk	Low risk	Low risk
Segni, MT et al	High risk	Low risk	Low risk	Low risk	Low risk	Low risk	Unclear	Low risk	Low risk	Low risk	Low risk
Azage M. et al	Low risk	Low risk	Low risk	Low risk	Low risk	Unclear	Low risk	Low risk	Low risk	Low risk	Low risk
Elshamy, Karima F et al	High risk	High risk	High risk	Low risk	Low risk	Low risk	Low risk	Low risk	Low risk	Low risk	Moderate risk
Akhigbe, A. O. et al	High risk	Low risk	Low risk	Low risk	Low risk	Unclear	Low risk	Low risk	Low risk	Low risk	Low risk
Nde et al.	High risk	Low risk	Low risk	Low risk	Low risk	Low risk	Low risk	Low risk	Low risk	Low risk	Low risk
Negeri et al.	Low risk	Low risk	Low risk	Low risk	Low risk	Unclear	Low risk	Low risk	Low risk	Low risk	Low risk
Odusanya et al	Low risk	Low risk	Low risk	Low risk	Low risk	Unclear	High risk	Low risk	Low risk	Low risk	Low risk
Ogunbode A M	High risk	High risk	High risk	Low risk	Low risk	Unclear	High risk	Low risk	Low risk	Low risk	High risk
Ossai EN et al.	High risk	Low risk	Low risk	Low risk	Low risk	Unclear	High risk	Low risk	Low risk	Low risk	Moderate risk
Feleke D. et al	Low risk	Low risk	Low risk	Low risk	Low risk	Low risk	Low risk	Low risk	Low risk	Low risk	Low risk
Kayode F.O. et al.	High risk	High risk	High risk	Low risk	Low risk	Unclear	Unclear	Low risk	Low risk	Low risk	High risk
Okobia, Michael N et al.	Low risk	Low risk	Low risk	Low risk	Low risk	Low risk	Low risk	Low risk	Low risk	Low risk	Low risk
Getu et al.	High risk	High risk	Low risk	Low risk	Low risk	Low risk	Low risk	Low risk	Low risk	Low risk	Low risk
Shallo et al.	High risk	Low risk	Low risk	Low risk	Low risk	Low risk	Low risk	Low risk	Low risk	Low risk	Low risk
Suh et al	Low risk	High risk	High risk	Low risk	Low risk	Low risk	Low risk	Low risk	Low risk	Low risk	Low risk
Ifediora, C. O., & Azuike, E. C.	High risk	Low risk	High risk	High Risk	Low risk	Low risk	Low risk	Low risk	Low risk	Low risk	Moderate risk
Ameer, K et al	High risk	High risk	High risk	Low risk	Low risk	Unclear	Low risk	Low risk	Low risk	Low risk	Moderate risk
Agboola AOJ et al	High risk	High risk	High risk	Low risk	Low risk	Low risk	Low risk	Low risk	Low risk	Low risk	Moderate risk
Amoran, O. E. and Toyobo, O. O	Low risk	Low risk	Low risk	Low risk	Low risk	Low risk	Low risk	Low risk	Low risk	Low risk	Low risk
Godfrey, Katende et al	Low risk	Low risk	Low risk	Low risk	Low risk	Low risk	Low risk	Low risk	Low risk	Low risk	Low risk
Bayumi E	High risk	High risk	High risk	Low risk	Low risk	Low risk	Unclear	Low risk	Low risk	Low risk	High risk
Bellgam H.I. amd Buowari Y. D	Low risk	Low risk	Low risk	Low risk	Low risk	Low risk	Low risk	Low risk	Low risk	Low risk	Low risk
Boulos, Dina NK and Ghali, Ramy R	High risk	High risk	High risk	Low risk	Low risk	Low risk	Low risk	Low risk	Low risk	Low risk	Moderate risk
E. Kudzawuet al.	Low risk	Low risk	Low risk	Low risk	Low risk	Low risk	Low risk	Low risk	Low risk	Low risk	Low risk
Fondjo LA et al	Low risk	Low risk	Low risk	Low risk	Low risk	Low risk	Low risk	Low risk	Low risk	Low risk	Low risk
Idris SA et al	High risk	High risk	High risk	Low risk	Low risk	Low risk	Low risk	Low risk	Unclear	Low risk	High risk
Kifle MM et al	Low risk	Low risk	Low risk	Low risk	Low risk	Low risk	Low risk	Low risk	Low risk	Low risk	Low risk
Morse EP et al	Low risk	Low risk	High risk	Low risk	Low risk	Low risk	Low risk	Low risk	Low risk	Low risk	Low risk
Ndikubwimana J et al	Low risk	Low risk	Low risk	Low risk	Low risk	Low risk	Low risk	Low risk	Low risk	Low risk	Low risk
Obaikol R et al	High risk	High risk	High risk	Low risk	Low risk	Low risk	Low risk	Low risk	Low risk	Low risk	Moderate risk
Ramathuba, Dorah U et al	Low risk	Low risk	Low risk	Low risk	Low risk	Low risk	Low risk	Low risk	Low risk	Low risk	Low risk
Ramson, Lombe Mumba	Low risk	Low risk	Low risk	Low risk	Low risk	Low risk	Low risk	Low risk	Low risk	Low risk	Low risk
Florence, Adeyemo O et al	Low risk	Low risk	High risk	Low risk	Low risk	Low risk	Low risk	Low risk	Low risk	Low risk	Low risk
Yakubu AA et al	Low risk	Low risk	High risk	Low risk	Low risk	Unclear	Low risk	Low risk	Low risk	Low risk	Low risk
Andegiorgishet al.	Low risk	Low risk	High risk	Low risk	Low risk	Low risk	Low risk	Low risk	Low risk	Low risk	Low risk
Kimani, SM and Muthumbi, E	High risk	High risk	High risk	Low risk	Low risk	Low risk	Low risk	Low risk	Low risk	Low risk	Moderate risk
Agbonifoh, Julia Adesua	Low risk	Low risk	High risk	Low risk	Low risk	Low risk	Low risk	Low risk	Low risk	Low risk	Low risk
Casmir, Ebirim Chikere Ifeanyi et al	Low risk	Low risk	Low risk	Low risk	Low risk	Low risk	Low risk	Low risk	Low risk	Low risk	Low risk
Joel Olayiwola Faronbi	Low risk	Low risk	Low risk	Low risk	Low risk	Low risk	Low risk	Low risk	Low risk	Low risk	Low risk
Makanjuola, OJ et al	Low risk	Low risk	Low risk	Low risk	Low risk	Low risk	Low risk	Low risk	Low risk	Low risk	Low risk
Olowokere et al.	Low risk	Low risk	Low risk	Low risk	Low risk	Low risk	Low risk	Low risk	Low risk	Low risk	Low risk
Sambo, MN et al	Low risk	Low risk	Unclear	Low risk	Low risk	Low risk	Low risk	Low risk	Low risk	Low risk	Low risk

### Data extraction

Data extraction of included articles was made using the Joanna Briggs Institute (JBI) tool for prevalence studies [25]. A Microsoft excel sheet was prepared and the following information were extracted; author/s name, title, year of publication, study area and country, study design, study setting, study population, age of the study participants, sample size, response rate, prevalence of breast self-examination practice (ever/regular).

### Heterogeneity and publication bias

The heterogeneity of included studies was assessed by using the I^2^ statistics. The *p*-value for I^2^ statistics less than 0.05 were used to determine the presence of heterogeneity. I^2^ values of 25, 50, and 75% are assumed to represent low, moderate and high heterogeneity respectively [26]. Graphically publication bias and small study effect were evaluated by funnel plot test. We had plotted the studies’ logit event rate and standard error to detect asymmetry in the distribution. When there is a gap in the funnel plot, it indicates that is a potential for publication bias. In addition, the publication bias was assessed using the Egger regression asymmetry test [27].

### Statistical analysis and synthesis

Findings were illustrated in the form of forest plots and tables. Eligible primary studies data were extracted, entered into Microsoft Excel and then exported to STATA version 13. Forest plot was used to present the combined estimate with 95% confidence interval (CI) of the meta analysis in Africa. The random effect model of analysis was used as a method of meta-analysis since it enables us to minimize the heterogeneity of included studies. Subgroup and sensitivity analyses were also conducted by different study characteristics such as sub-regions of Africa (East, South, West, Central and Northern Africa), study period (2000–2005, 2006–2010, 2011–2015, 2016–2020), setting (community/institution based), study area (urban, rural or both), study participants’ profession (health/non health professionals), and risk of bias (low, moderate and high).

## Result

### Characteristics of included studies

A total of 56 studies were included in this meta-analysis. Fourteen African countries were included in this review. From the included studies, 25(44.64%) were from West Africa [28–52], 22(39.29%) from East Africa [19, 53–73], 5(8.93%) from North Africa [21, 74–77], 3(5.36%) from Central Africa [78, 79], 1(1.79%) from South Africa [80]. All the included fifty-six studies in this systematic review and meta-analysis conducted in African countries were cross sectional study designs.

The sample size of the included studies ranged from a minimum of 100 in a study conducted in Nigeria [29, 49, 50] to a maximum of 1036 a study conducted in Ghana [44]. A total of 19, 228 study participants were included in this review (Table 2). Almost all 55(98.21%) of the included studies were published on peer reviewed journals while only 1(1.178%) study was unpublished [58]. Majority 43(76.79%) of the included studies were institution based while around one forth 13(23.21%) of the studies were community based [19, 28, 30, 38, 41–43, 50, 51, 62, 71, 80, 81]. From the total included studies, 10(17.86%) were conducted among health professionals [19, 33, 40, 42, 46, 54, 61, 64, 72, 75]. Majority 40 (71.43%) of the study participant were urban residents and the age of the participants ranged from 13 [32] to 85 [42] year-old.

**Table 2:** Summary of characteristics of included studies in meta-analysis of breast self-examination practice in Africa

Author/s	Year	Sub- region	Study design	Study setting	Response rate	Sample size	Event (Ever Practiced)	Prevalence of BSE (%)		Risk of Bias
								Ever BSE	Regular BSE	
Birhane et al.	2015	East Africa	Cross sectional	Institution based	99.6	315	38	12	Not reported	Low risk
Obaji et al.	2013	West Africa	Cross sectional	Community Based	100	238	52	21.8	0.24	Moderate risk
Onwere et al.	2009	West Africa	Cross sectional	Institution based	100	100	78	78	78	Moderate risk
Abay et al.	2018	East Africa	Cross sectional	Institution based	99	404	26	6.4	6.2	Low risk
Minasie A et al.	2017	East Africa	Cross sectional	Institution based	100	281	128	46.5	6.4	Low risk
Abdel Fattah, M et al.	2000	North Africa	Cross sectional	Institution based	100	565	59	10.4	2.7	Moderate risk
Abeje et al.	2019	East Africa	Cross sectional	Institution based	100	633	154	24.3	10.1	Low risk
Birhane K et al.	2017	East Africa	Cross sectional	Institution based	94	400	113	28.3	17.5	Low risk
Sama, C. B. et al	2017	Central Africa	Cross sectional	Institution based	82.1	345	133	38.5	Not reported	Low risk
Kasahun AF	2014	East Africa	Cross sectional	Institution based	95.2	400	62	15.5	9.25	Low risk
Dagne AH et al.	2019	East Africa	Cross sectional	Institution based	100	421	137	32.5	15.2	Low risk
Dadzi R, Adam A	2019	West Africa	Cross sectional	Community Based	100	385	106	27.5	16.1	Low risk
Gwarzo, UMD et al	2009	West Africa	Cross sectional	Institution based	100	221	126	57	19	Low risk
Isara, A. R. and Ojedokun, C. I	2011	West Africa	Cross sectional	Institution based	95.7	287	29	10.1	Not reported	Low risk
Segni, MT et al	2016	East Africa	Cross sectional	Institution based	100	368	145	39.4	2.3	Low risk
Azage M. et al	2013	East Africa	Cross sectional	Community Based	98.01	395	147	32.2	14.2	Low risk
Elshamy, Karima F et al	2010	North Africa	Cross sectional	Institution based	80	133	75	56.4	10.5	Moderate risk
Akhigbe, A. O. et al	2009	West Africa	Cross sectional	Institution based	77.8	393	305	77.6	Not reported	Low risk
Nde et al.	2015	Central Africa	Cross sectional	Institution based	91.1	166	62	37.3	3	Low risk
Negeri et al.	2017	East Africa	Cross sectional	Institution based	95.5	300	231	77	33.7	Low risk
Odusanya et al	2001	West Africa	Cross sectional	Institution based	94	188	167	88.9	61.7	Low risk
Ogunbode A M	2015	West Africa	Cross sectional	Institution based	100	140	87	62	7.9	High risk
Ossai EN et al.	2019	West Africa	Cross sectional	Institution based	100	365	232	63.6	15.9	Moderate risk
Feleke D. et al	2019	East Africa	Cross sectional	Community Based	100	810	70	8.6	Not reported	Low risk
Kayode F.O. et al.	2005	West Africa	Cross sectional	Institution based	84	341	181	53	33.7	High risk
Okobia, Michael N et al.	2006	West Africa	Cross sectional	Community Based	95.1	1000	349	34.9	Not reported	Low risk
Getu et al.	2019	East Africa	Cross sectional	Institution based	100	407	87	21.4	11	Low risk
Shallo et al.	2019	East Africa	Cross sectional	Institution based	87.9	340	163	47.9	32.4	Low risk
Suh et al	2012	Central Africa	Cross sectional	Community Based	100	120	72	60	Not reported	Low risk
Ameer, K et al	2014	East Africa	Cross sectional	Institution based	100	126	29	23	Not reported	Moderate risk
Ifediora, C. O., & Azuike, E. C.	2018	West Africa	Cross sectional	Institution based	74.3	321	148	46.1	6.2	Moderate risk
Agboola AOJ et al	2009	West Africa	Cross sectional	Institution based	100	115	98	85.2	46.9	Moderate risk
Amoran, O. E. and Toyobo, O. O	2015	West Africa	Cross sectional	Community Based	–	495	121	24.4	5.23	Low risk
Godfrey, Katende et al	2016	East Africa	Cross sectional	Institution based	100	204	89	43.6	19.6	Low risk
Bayumi E	2016	North Africa	Cross sectional	Institution based	100	240	91	37.9	15.8	High risk
Bellgam H.I. amd Buowari Y. D	2012	West Africa	Cross sectional	Community Based	98.7	691	200	28.9	Not reported	Low risk
Boulos, Dina NK and Ghali, Ramy R	2013	North Africa	Cross sectional	Institution based	89.8	543	40	7.4	1.3	Moderate risk
E. Kudzawuet al.	2016	West Africa	Cross sectional	Community Based	100	170	132	77.6	68	Low risk
Fondjo LA et al	2018	West Africa	Cross sectional	Institution based	100	1036	831	80.2	8.1	Low risk
Idris SA et al	2013	North Africa	Cross sectional	Institution based	88.9	200	129	64.5	64.5	High risk
Kifle MM et al	2016	East Africa	Cross sectional	Institution based	100	380	51	13.4	5.5	Low risk
Morse EP et al	2014	East Africa	Cross sectional	Institution based	100	225	75	33.3	14.2	Low risk
Ndikubwimana J et al	2016	East Africa	Cross sectional	Institution based	94.8	229	55	24	4.4	Low risk
Obaikol R et al	2010	East Africa	Cross sectional	Institution based	98.1	314	96	30.6	14	Moderate risk
Ramathuba, Dorah U et al	2015	South Africa	Cross sectional	Community Based	100	150	8	5.3	0	Low risk
Ramson, Lombe Mumba	2017	East Africa	Cross sectional	Community Based	100	351	99	28.2	12	Low risk
Florence, Adeyemo O et al	2016	West Africa	Cross sectional	Institution based	100	200	200	100	75	Low risk
Yakubu AA et al	2014	West Africa	Cross sectional	Institution based	100	102	93	91.1	44.1	Low risk
Andegiorgishet al.	2018	East Africa	Cross sectional	Institution based	97	414	313	75.6	45.9	Low risk
Kimani, SM and Muthumbi, E	2008	East Africa	Cross sectional	Institution based	100	169	114	67.5	20.1	Moderate risk
Agbonifoh, Julia Adesua	2016	West Africa	Cross sectional	Institution based	93.2	647	397	61.4	18.7	Low risk
Casmir, Ebirim Chikere Ifeanyi et al	2015	West Africa	Cross sectional	Institution based	100	720	552	76.7	32.5	Low risk
Joel Olayiwola Faronbi	2012	West Africa	Cross sectional	Institution based	100	100	82	82	12	Low risk
Makanjuola, OJ et al	2013	West Africa	Cross sectional	Community Based	100	100	25	25	13	Low risk
Olowokere et al.	2012	West Africa	Cross sectional	Community Based	100	180	49	27.2	Not reported	Low risk
Sambo, MN et al	2013	West Africa	Cross sectional	Institution based	100	345	189	54.8	13.9	Low risk

### Prevalence of breast self-examination practice in Africa

**Fig. 2:**
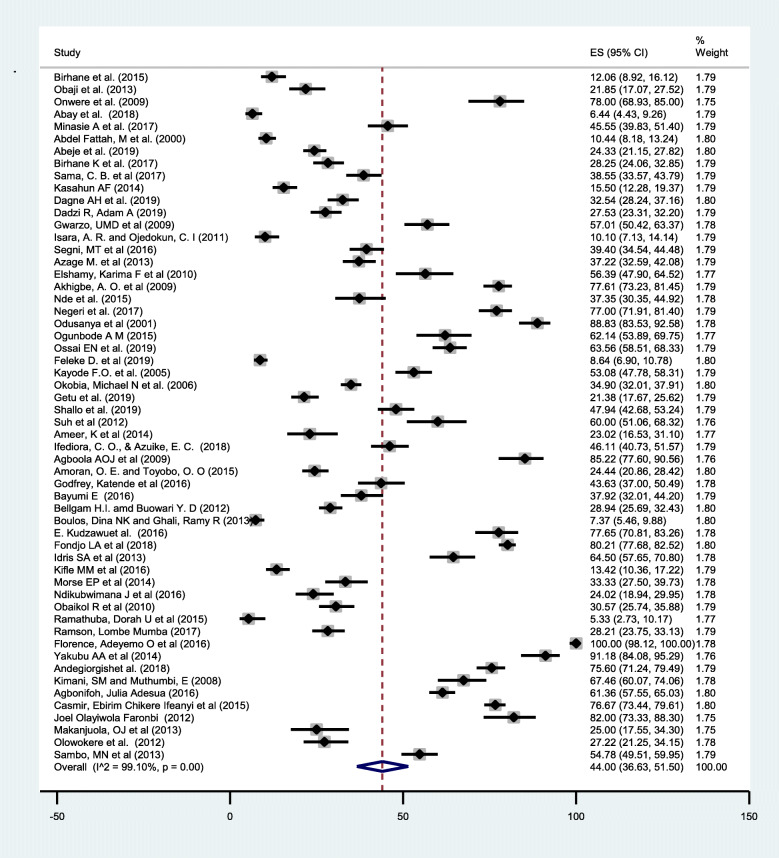
Forest plot displaying the pooled prevalence of ever breast self-examination practice among women in Africa

**Fig. 3:**
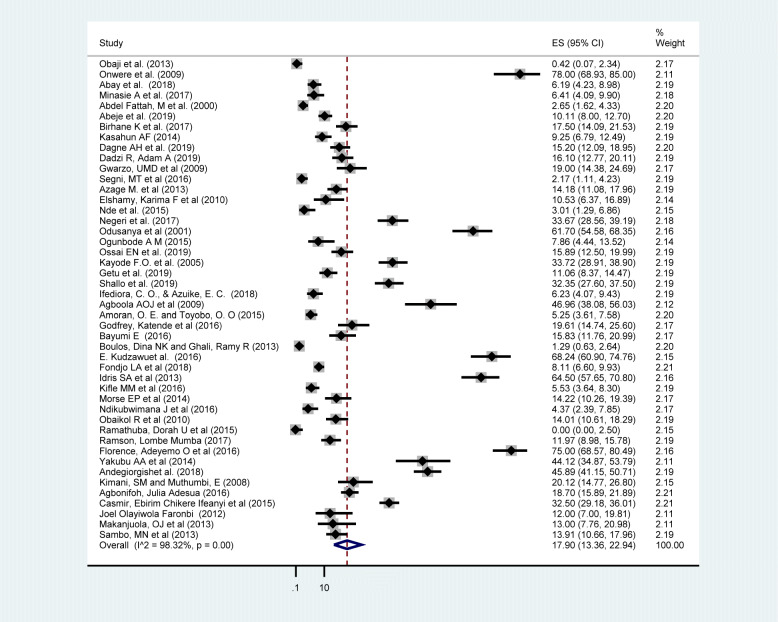
Forest plot displaying the pooled prevalence of regular breast self-examination practice among women in Africa

The pooled prevalence of ever breast self-examination practice in Africa was 44.0% (95% CI: 36.63, 51.50) (Fig. 2). Whereas the pooled prevalence of regular breast self-examination practice was 17.9% (95% CI: 13.36, 22.94) (Fig. 3). The lowest breast self-examination was reported in South Africa 5.3% (95% CI: 2.73, 10.17) [80] and the highest was in Nigeria 100%(95% CI: 98.12, 100.00) [45]. The prevalence of breast self-examination was highest 58.87% (95% CI: 48.06, 69.27) in West Africa followed by Central Africa 44.87% (95% CI: 32.50, 57.57), North Africa 32.63%(95% CI: 12.09–57.46), East Africa 32.18%(95%CI: 23.74,41.24) and the lowest was in South Africa 5.33% (95% CI: 2.73,10.17). The I-square test result showed that there was a high heterogeneity among the included studies (I^2^ = 99.10%, *p-*value = < 0.001). This result is an indicative to use the random effect model and subgroup analysis.

### Subgroup analysis

A subgroup analysis was conducted since there was statistically significant heterogeneity, I-square test statistics less than 0.05(I^2^ = 99.10%, *p-*value = < 0.001). The purpose of the analysis was to identify the source of heterogeneity so that correct interpretation of the findings is made. We did subgroup meta-analysis of the included studies by sub region, study setting, study period, study participants, place of resident and risk of bias. However, the subgroup analysis found no significant variable which can explain the heterogeneity in this review. Therefore, the heterogeneity can be explained by other factors not included in this review.

**Fig. 4:**
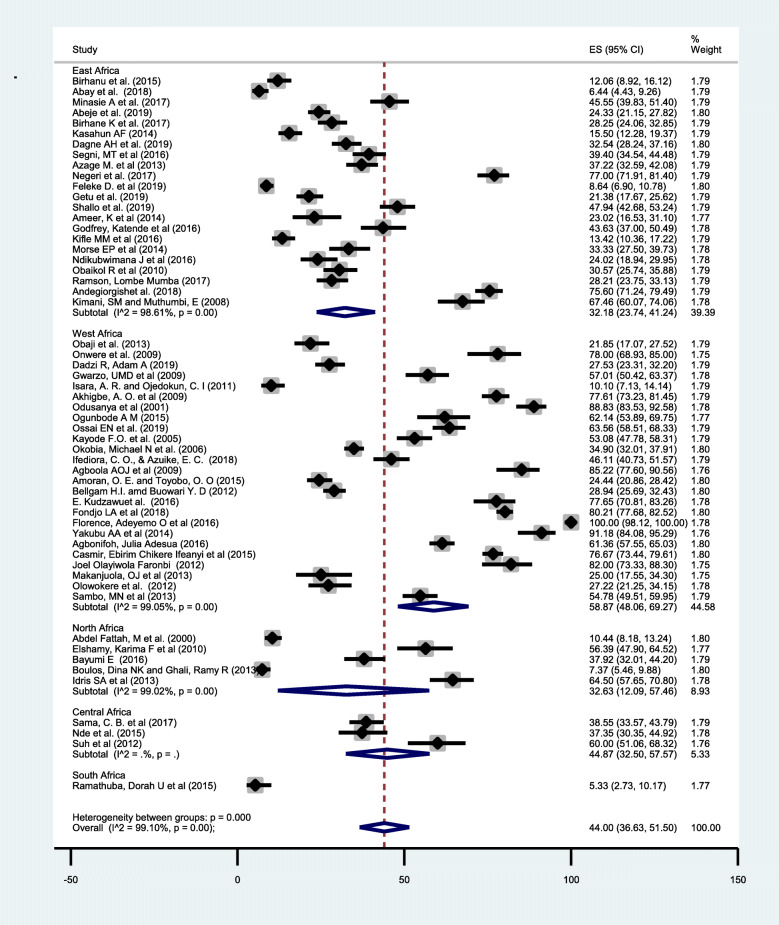
Forest plot of ever breast self-examination practice in Africa by sub region

The highest prevalence of ever breast self-examination practice was reported in West African countries 58.87% (95%CI: 48.06,69.27) while the lowest was in South African country’s 5.33% (95%CI: 2.73,10.17) (Fig. 4). A higher 48.39%(95%CI:39.39,57.44) prevalence of breast self-examination among institutional based studies compared with community-based studies 29.95% (95%CI:21.53, 39.11). In the subgroup analysis by publication period there was irregular trend in the practice of breast self-examination practice. The highest, 61.42% (95%CI:45.28, 76.39) prevalence of breast self-examination practice was reported during 2006–2010 while the lowest, 38.58% (95%CI: 27.39, 50.42) was in the period of 2011–2015. Breast self-examination practice was higher 63.33% (95% CI: 48.62, 76.88) among health professionals and urban residents 48.55% (95% CI:39.20,57.95). The prevalence of breast self-examination among low risk of bias studies was 43.20% (95%CI: 34.53, 52.08) and 54.30 (95%CI: 42.62,65.75) for high risk of bias studies (Table 3).

**Table 3:** Subgroup analysis of the prevalence of breast self-examination practice in Africa

Subgroup		Number of studies	Prevalence BSE Practice (95% CI)	Heterogeneity	
				I^**2**^	***p***-value
**Sub region**	West Africa	25	58.87(48.06, 69.27)	99.05	< 0.001
	East Africa	22	32.18 (23.74, 41.24)	98.61	< 0.001
	North Africa	5	32.63(12.09, 57.46)	99.02	< 0.001
	Central Africa	3	44.87(32.50, 57.57)	–	–
	South Africa	1	5.33 (2.73,10.17)	–	–
**Study participant**	Health professional	10	63.33(48.62, 76.88)	98.56	< 0.001
	Non health professionals	46	39.81(31.85, 48.06)	99.12	< 0.001
**Study setting**	Institutional based	43	48.39(39.39,57.44)	99.16	< 0.001
	Community based	13	29.95(21.53, 39.11)	97.85	< 0.001
**Publication Period**	2000–2005	3	50.50(8.05, 92.48)	–	–
	2006–2010	8	61.42(45.28, 76.39)	98.28	< 0.001
	2011–2015	22	38.58(27.39, 50.42)	98.88	< 0.001
	2016–2020	23	42.34 (30.75, 54.37)	99.29	< 0.001
**Risk of bias**	Low	41	43.20(34.53, 52.08)	99.19	< 0.001
	Moderate	11	43.26 (26.29, 61.07)	98.95	< 0.001
	High	4	54.30 (42.62,65.75)	92.04	< 0.001
**Place of residence**	Urban	40	48.55(39.20,57.95)	99.18	< 0.001
	Rural	12	34.25(23.60, 45.75)	98.36	< 0.001
	Mixed	4	28.78(15.04, 44.86)	97.15	< 0.001
**Total**		**56**	**44.0% (36.63, 51.50)**	**99.10**	**< 0.001**

### Sensitivity analysis

Sensitivity analysis was done to assess the effect of each study on the heterogeneity by excluding studies with small sample size (n < =100) and high risk of bias one by one. However, the excluded studies did not brought reduction in the heterogeneity of the estimates (Table 4).

**Table 4:** Sensitivity analysis of the included studies to estimate the pooled prevalence of breast self-examination practice among women in Africa

S. No	Study Omitted	Reason for omission	Pooled prevalence of BSE practice (95% CI)	I^2^ values
1.	Ogunbode A M, 2015	High risk of bias	43.67(36.24–51.2)	99.10
2.	Kayode F.O. et al., 2005	High risk of bias	43.84 (36.35–51.46)	99.11
3.	Bayumi E et al., 2016	High risk of bias	44.11(36.63–51.73)	99.12
4.	Idris SA et al., 2013	High risk of bias	43.63(36.20–51.21)	99.11
5.	Onwere et al., 2009	Small sample size (100)	43.37(35.98–50.92	99.11
6.	Joel Olayiwola Faronbi et, 2012	Small sample size (100)	43.28(35.90–50.82)	99.11
7.	Makanjuola, OJ et al., 2013	Small sample size (100)	44.36(36.90–51.94)	99.12

### Risk of bias

Studies included in this meta-analysis were assessed for risk of bias by using Hoy 2012 tool [24] (Table 1). From the 56 included studies, 41(73.21%) of them were categorized as low risk [19, 30–33, 38, 41–53, 55–64, 66–69, 71, 72, 78–83], 11(19.64%) moderate risk [28, 29, 36, 39, 40, 65, 70, 73–75, 77] and 4(7.14%) high risk of bias [21, 35, 37, 76]. It is also found that 23(41.1%) and 21(37.5%) of the included studies did not apply random selection and represent the national population respectively.

### Publication bias

**Fig. 5:**
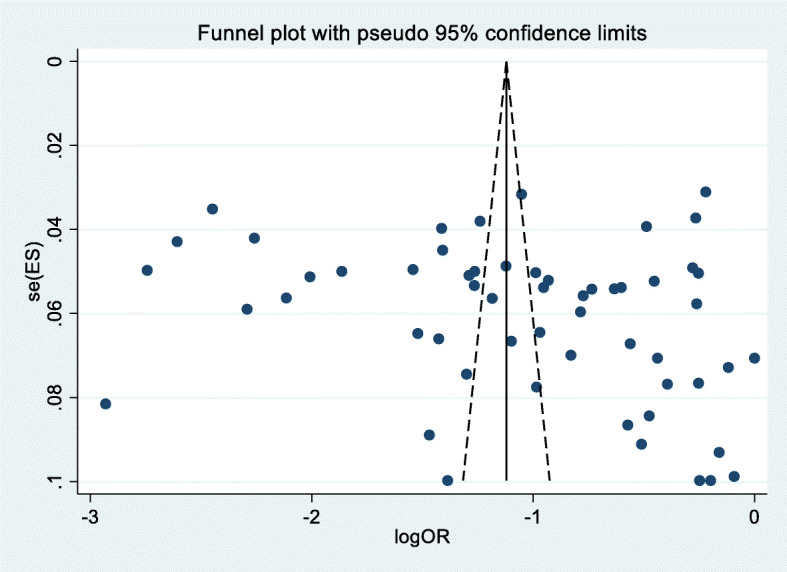
Graphic representation of publication bias using funnel plot of included studies in systematic review and meta-analysis of breast self-examination practice among women in Africa

Small study effect of the included studies was assessed through visually and statistically. In this meta-analysis there was no publication bias since the included studies were distributed symmetrically in the funnel plot (Fig. 5). Additionally, the result of Egger’s test showed that no publication bias (*p-* value = 0.232).

## Discussion

In low and middle income countries, breast self-examination is one of feasible and practical options to screen breast cancer at an early stage [84, 85]. Breast self-examination has shown in reduction of incidence and death, improvement of survival rate and detection of breast cancer at an early stage [86, 87]. This systematic review and meta-analysis is paramount in showing the status of breast self-examination practice in Africa. This review showed that significant numbers of women in Africa are not practicing breast examination.

In this meta-analysis the overall pooled prevalence of ever breast self-examination practice was 44.0% (95%CI: 36.63, 51.50). The finding was comparable (44.4%) with a study conducted in Indonesia [88] among women in the age group of 20–60. However, it is higher than a nationwide cancer screening survey in South Korea (16.1%) [89] and Russia (24%) [90]. This discrepancy might be attributed due to difference in the age of the study population. In this meta-analysis majority (67.9%) of the study participant are younger age groups [20–40] and this age groups are more likely to perform breast self-examination than older one [91]. On the other hand, this finding was lower than a study conducted among nurses in Poland (100%) [91] and University staffs in Malaysia 83.7% [92]. This discrepancy might be attributed due to difference in the study population as health professionals and university staffs are more aware and skilled about breast self-examination compared to the general population.

The pooled prevalence of regular (monthly) breast self-examination practice was 17.9% (95% CI: 13.36, 22.94) which is comparable (15.2%) with a study done in Vietnam [93]. However, the finding was lower than a study done in Poland (56.7%) [91], Malaysia (41%) [92], Russia (32%) [90]. This might be attributed due to difference in culture and tradition towards breast self-examination in the study population. In addition to this, the level of awareness and information dissemination about breast self-examination frequency and interval is not well addressed in African women compared to European and Asian. This indicates that even if breast self-examination is the most feasible and affordable option to early diagnose breast cancer, African women are not practicing as per the recommended frequency and interval.

In the sub group analysis, the highest prevalence of ever breast self-examination practice was reported in West African countries 58.87% (95%CI: 48.06, 69.27) compared with other regions. The possible reason for this variation might be attributed due to the difference in the study population. In this review, 25 studies were included from West African region and among this 17(68%) of the studies were conducted among urban residents. In general, urban resident tends to have positive attitudes toward and as well as better awareness about breast self-examination. Breast self-examination practice was higher 63.33% (95% CI: 48.62, 76.88) among health professionals compared with non-health professionals. This might be attributed to the level of awareness about the disease, skill difference to perform the procedure and perception towards breast self-examination practice. Additionally, health care providers are expected to be role models for other women and because of this reason they engaged more in breast self-examination.

### Limitation of the study

The estimation of the pooled prevalence of breast self-examination may have been affected by the heterogeneity, as suggested by the very high I^2^ statistic of 99.10%. This might be attributed to the methodological variation among the included studies. We have also included only articles published in English language and some of the included articles published on emerging journals. Some of the studies included in this review had small sample size and this might affect the pooled estimate finding. Furthermore, most of the studies included in this meta-analysis were represented from west and east African countries due to the limited number of studies in the other areas. Therefore, some regions may be underrepresented.

## Conclusion

### Implications for practice

This systematic review and meta-analysis found that the pooled prevalence of ever and regular breast self-examination was very low compared with other LMIC and high income countries. Even though, most literatures recommend regular breast self-examination is feasible and practical screening options for LMIC nations, the practice was not satisfactory in Africa. Therefore, intensive behavioral change communication and interventions that emphasize different domains should be given by stakeholders to increase the practice of breast self-examination in Africa.

### Implications for research

In low and middle income countries breast self-examination is a feasible and beneficial approach to reduce morbidity and mortality of breast cancer through early diagnosis. Thus, further large scale follow-up studies should be conducted to identify barriers and challenges of breast self-examination practice among women in Africa.

## Supplementary Information

**Additional file 1.**

**Additional file 2.**

## Data Availability

All data pertaining to this review were included and presented in the document as well its supplementary files.

## Abbreviations

BSE: Breast self-examination practice
CBE: Clinical breast examination
CI: Confidence interval
GLOBOCAN: Global Cancer Observatory
JBI-MAStARI: Joanna Briggs Institute Meta-Analysis of Statistics Assessment and Review Instrument
LMIC: Low and Middle Income Countries
PRISMA: Preferred Reporting Items of Systematic Reviews and Meta-Analysis
SE: Standard error
SDG: Sustainable Development Goal
