# Supplementary material for: Breast self-examination practice among women in Africa: a systematic review and Meta-analysis
Source: Arch Public Health. 2021 Aug 21;79:149. doi: 10.1186/s13690-021-00671-8 (PMC8379892; doi:10.1186/s13690-021-00671-8)
Supplement: Supplementary file 2 — Additional file 2. [file 13690_2021_671_MOESM2_ESM.docx]

**Supplementary file 2.** **Detailed search strategy for database searches**

| **No.** | **Databases (Total 8)** | **Search Terms** | **Results**  **Total = 829** |
| --- | --- | --- | --- |
|  | PubMed | ((("Breast Self Examination"[MeSH Terms] OR ("Breast Self Examination"[All Fields] OR "self examination breast"[All Fields] OR "Breast Self-Examinations"[All Fields] OR "self examination breast"[All Fields] OR "self examinations breast"[All Fields] OR "early detection of breast cancer"[All Fields] OR "breast cancer screening"[All Fields])) AND ("health knowledge, attitudes, practice"[MeSH Terms] OR "practice"[All Fields] OR "practice s"[All Fields] OR "practiced"[All Fields] OR "practices"[All Fields] OR "practicing"[All Fields])) AND ("women"[MeSH Terms] OR ("Girls"[All Fields] OR "Girl"[All Fields] OR "Woman"[All Fields] OR "female"[All Fields] OR "females"[All Fields] OR "Reproductive age women"[All Fields] OR "reproductive aged women"[All Fields])) AND ("Africa"[MeSH Terms] OR ((("Africa central"[Title/Abstract] OR "Cameroon"[Title/Abstract] OR "Central African Republic"[Title/Abstract] OR "Chad"[Title/Abstract] OR "Congo"[Title/Abstract] OR "Democratic Republic of the Congo"[Title/Abstract] OR "Equatorial Guinea"[Title/Abstract] OR "Gabon"[Title/Abstract] OR "Sao Tome"[Title/Abstract] OR "Africa eastern"[All Fields]) AND "Burundi"[Title/Abstract]) OR "Djibouti"[Title/Abstract] OR "Eritrea"[Title/Abstract] OR "Ethiopia"[Title/Abstract] OR "Kenya"[Title/Abstract] OR "Rwanda"[Title/Abstract] OR "Somalia"[Title/Abstract] OR "South Sudan"[Title/Abstract] OR "Sudan"[Title/Abstract] OR "Tanzania"[Title/Abstract] OR "Uganda"[Title/Abstract] OR "Africa southern"[Title/Abstract] OR "Angola"[Title/Abstract] OR "Botswana"[Title/Abstract] OR "Eswatini"[Title/Abstract] OR "Lesotho"[Title/Abstract] OR "Malawi"[Title/Abstract] OR "Mozambique"[Title/Abstract] OR "Namibia"[Title/Abstract] OR "South Africa"[Title/Abstract] OR "Zambia"[Title/Abstract] OR "Zimbabwe"[Title/Abstract] OR "Africa western"[Title/Abstract] OR "Benin"[Title/Abstract] OR "Burkina Faso"[Title/Abstract] OR "Cabo Verde"[Title/Abstract] OR "Cote d'Ivoire"[Title/Abstract] OR "Gambia"[Title/Abstract] OR "Ghana"[Title/Abstract] OR "Guinea"[Title/Abstract] OR "Guinea-Bissau"[Title/Abstract] OR "Liberia"[Title/Abstract] OR "Mali"[Title/Abstract] OR "Mauritania"[Title/Abstract] OR "Niger"[Title/Abstract] OR "Nigeria"[Title/Abstract] OR "Senegal"[Title/Abstract] OR "Sierra Leone"[Title/Abstract] OR "Togo"[Title/Abstract] OR "Africa northern"[Title/Abstract] OR "Algeria"[Title/Abstract] OR "Egypt"[Title/Abstract] OR "Libya"[Title/Abstract] OR "Morocco"[Title/Abstract] OR "Tunisia"[Title/Abstract])) | **133** |
|  | Cochrane | ("Breast Self Examination" OR "Self-Examination, Breast" OR “Breast Self-Examinations" OR "Self Examination, Breast" OR "Self-Examinations, Breast" OR “early detection of breast cancer” OR “breast cancer screening”) AND "health knowledge OR attitudes OR practice/s AND ("Girls" OR " Girl" OR "Woman" OR "female” OR "females" OR “Reproductive age women” OR “Reproductive - aged women”) AND (Africa) | **0** |
|  | EMBASE | ("Breast Self Examination" OR "Self-Examination, Breast" OR “Breast Self-Examinations" OR "Self Examination, Breast" OR "Self-Examinations, Breast" OR “early detection of breast cancer” OR “breast cancer screening”) AND "health knowledge OR attitudes OR practice/s AND ("Girls" OR " Girl" OR "Woman" OR "female” OR "females" OR “Reproductive age women” OR “Reproductive - aged women”) AND (Africa) | **0** |
|  | Science Direct | ("Breast Self Examination" OR "Self-Examination, Breast" OR “Breast Self-Examinations" OR "Self Examination, Breast" OR "Self-Examinations, Breast" OR “early detection of breast cancer” OR “breast cancer screening”) AND "health knowledge OR attitudes OR practice/s AND ("Girls" OR " Girl" OR "Woman" OR "female” OR "females" OR “Reproductive age women” OR “Reproductive - aged women”) AND (Africa) | **11** |
|  | HINARI | ("Breast self examination") AND (“health knowledge” OR attitudes OR practice) AND (women) AND (Africa) | **387** |
|  | African Journals Online (AJOL) | ("Breast Self Examination" OR "Self-Examination, Breast" OR “Breast Self-Examinations" OR "Self Examination, Breast" OR "Self-Examinations, Breast" OR “early detection of breast cancer” OR “breast cancer screening”) AND "health knowledge OR attitudes OR practice/s AND ("Girls" OR " Girl" OR "Woman" OR "female” OR "females" OR “Reproductive age women” OR “Reproductive - aged women”) AND (Africa) | **209** |
|  | WHO Global Index Medicus | ("Breast Self Examination" OR "Self-Examination, Breast" OR “Breast Self-Examinations" OR "Self Examination, Breast" OR "Self-Examinations, Breast" OR “early detection of breast cancer” OR “breast cancer screening”) AND "health knowledge OR attitudes OR practice/s AND ("Girls" OR " Girl" OR "Woman" OR "female” OR "females" OR “Reproductive age women” OR “Reproductive - aged women”) AND (Africa) | **2** |
|  | Google Scholar | Where my words occur: in the title of the article: 1. With all of the words: Breast Self-Examination With at least one of the words: "Breast Self Examination, Self-Examination, Breast, Breast Self-Examinations, Self Examination, Breast, Self-Examinations, Breast, early detection of breast cancer, breast cancer screening”  2. With all of the words: Practice  With at least one of the words: health knowledge, attitudes, practiced, practices, practicing 3. With all of the words: Women  With at least one of the words: Girls, Girl, Woman, female, females, Reproductive age women “Reproductive - aged women”  4. With all of the words: Africa  With at least one of the words: Africa, Central, Africa, Eastern, Africa, Southern, Africa, Western, Africa, Northern | **86** |
|  | Unpublished Thesis work | ("Breast self examination") AND (“health knowledge” OR attitudes OR practice) AND (women) AND (Africa) | **1** |
